# Omega-3 Supplementation Modulates Whole-Blood EPA and DHA Without Improving Patient-Reported Outcomes in Youth with Migraine: A Quasi-Randomized Pilot Trial

**DOI:** 10.3390/nu18182983

**Published:** 2026-09-11

**Authors:** Samantha Glover, Anne E. Sanders, Daisy Zamora, Keturah R. Faurot, Saame Raza Shaikh, Kimon Divaris, Klaus Werner, Steven P. Trau, Jaclyn Bain, Jialiu Xie, Sydney Danze, Sajan Singh, Thomas Southern, Joseph Iskander, Caroline M. Sawicki

**Affiliations:** 1 Department of Pediatric Dentistry and Dental Public Health, Adams School of Dentistry, University of North Carolina, Chapel Hill, NC 27599, USA; sag40@email.unc.edu (S.G.); anne_sanders@unc.edu (A.E.S.); kimon_divaris@unc.edu (K.D.); jacbain@unc.edu (J.B.); sydney15@live.unc.edu (S.D.); sajans@unc.edu (S.S.); trsouth@unc.edu (T.S.); jjoseph1@live.unc.edu (J.I.); 2 Department of Physical Medicine and Rehabilitation, School of Medicine, University of North Carolina, Chapel Hill, NC 27514, USAfaurot@med.unc.edu (K.R.F.); 3 Department of Nutrition, Gillings School of Global Public Health and School of Medicine, University of North Carolina, Chapel Hill, NC 27599, USA; shaikhsa@email.unc.edu; 4 Department of Epidemiology, Gillings School of Global Public Health, University of North Carolina, Chapel Hill, NC 27599, USA; 5 Division of Pediatric Neurology, Duke University Medical Center, Durham, NC 27705, USA; klaus.werner@duke.edu; 6 Department of Neurology, School of Medicine, University of North Carolina, Chapel Hill, NC 27599, USA; steven_trau@neurology.unc.edu; 7 Department of Biostatistics, Gillings School of Public Health, University of North Carolina, Chapel Hill, NC 27599, USA; jialiux2@live.unc.edu

**Keywords:** omega-3 fatty acids, pediatric migraine, lipid biomarkers, dietary supplementation

## Abstract

Background/Objectives: Omega-3 polyunsaturated fatty acids (PUFAs) influence lipid-mediated inflammatory pathways implicated in migraine, yet randomized trial data evaluating biologic effects of omega-3 supplementation in youth with migraine remain limited. This pilot quasi-randomized clinical trial aimed to determine whether 12 weeks of omega-3 PUFA supplementation produces measurable changes in whole-blood EPA and DHA levels in children and adolescents with migraine, and to characterize changes in patient-reported measures of pain, migraine-related disability, and psychological distress. Methods: We conducted a parallel-group, quasi-randomized, double-blind, placebo-controlled trial in children and adolescents (ages 10–17) with migraine diagnosed per International Classification of Headache Disorders, 3rd edition criteria, recruited from pediatric neurology clinics at two academic medical centers. Of 57 participants allocated 1:1 to daily omega-3 PUFA supplementation (340 mg EPA + 510 mg DHA) or coconut oil placebo for 12 weeks, 44 completed the study and were analyzed. The primary outcome was change in whole-blood omega-3 PUFA index. Secondary outcomes included patient-reported measures of pain intensity, pain interference, migraine-related disability, and psychological distress. Exploratory outcomes included changes in omega-6/omega-3 and arachidonic acid/eicosapentaenoic acid (AA/EPA) ratios. Between-group differences were evaluated using ANCOVA adjusting for baseline values. Results: Omega-3 supplementation significantly altered lipid biomarker profiles. Compared with placebo, the intervention group demonstrated greater increases in omega-3 PUFA index (adjusted mean difference 1.64; 95% CI 0.94–2.35; *p* < 0.001) and greater reductions in omega-6/omega-3 ratio (−2.57; 95% CI −4.25 to −0.90; *p* = 0.003) and AA/EPA ratio (−13.52; 95% CI −21.55 to −5.49; *p* = 0.002), consistent with the increase in whole-blood omega-3 levels. No significant between-group differences were observed in patient-reported outcomes. Conclusions: Omega-3 PUFA supplementation significantly altered lipid biomarker profiles in youth with migraine over 12 weeks, supporting a measurable whole-blood EPA + DHA response in this proof-of-concept trial. Future larger-scale trials with longer duration are needed to evaluate clinical effects.

## 1. Introduction

Migraine is among the most common neurological disorders affecting children and adolescents, with an estimated prevalence of approximately 11% [1] and increasing global burden over recent decades. Early-onset migraine frequently persists into adulthood and is associated with long-term disability [2], yet evidence-based treatment options for this age group remain limited. Most randomized controlled trials evaluating pharmacologic prophylaxis in children and adolescents have failed to demonstrate superiority over placebo, with placebo response rates ranging from 30% to 61% [3,4]. A landmark trial found that neither topiramate nor amitriptyline was superior to placebo for migraine prevention in patients aged 8–17 years, with an unfavorable risk-benefit profile for both agents [5]. Network meta-analyses have confirmed that few pharmacologic treatments demonstrate robust efficacy in this population, and those that do are associated with increased rates of adverse events [2,6]. These limitations, combined with challenges related to long-term adherence and tolerability in pediatric populations, have contributed to growing interest in complementary and nutrition-based approaches for migraine management [4].

Omega-3 polyunsaturated fatty acids (PUFAs), including eicosapentaenoic acid (EPA) and docosahexaenoic acid (DHA), are conditionally essential fatty acids with anti-inflammatory, anti-nociceptive, antioxidant, and neuromodulatory properties [7]. EPA and DHA can be obtained directly from marine dietary sources (e.g., fatty fish) or synthesized endogenously from the plant-derived essential fatty acid alpha-linolenic acid (ALA) through sequential desaturation and elongation steps; however, this conversion is highly inefficient in humans, making direct dietary intake the primary determinant of tissue EPA and DHA status [8,9]. Recommended daily EPA + DHA intake has not been established for children and adolescents, and population data indicate that most U.S. youth consume levels well below those proposed for optimal health [10,11]. Neuroinflammation is increasingly recognized as a key event in migraine pathophysiology, involving activation and sensitization of the trigeminovascular system, release of neuropeptides such as calcitonin gene-related peptide (CGRP), and production of pro-inflammatory lipid mediators [7,12]. At the cellular level, EPA and DHA modulate neuroinflammation through multiple mechanisms, including inhibition of microglial activation, suppression of pro-inflammatory cytokine release (e.g., tumor necrosis factor-alpha (TNF-α, interleukin (IL)-1 beta (β, IL-6) and production of specialized pro-resolving mediators (SPMs) such as resolvins, protectins, and maresins that actively promote resolution of inflammation and reduce nociceptive signaling [13,14]. In adults with migraine, dietary enrichment with EPA and DHA shifted circulating oxylipin profiles toward antinociceptive pathways and reduced headache frequency and disability [15,16], although a meta-analysis of earlier trials found no significant effect on migraine frequency or severity [17].

Pediatric data on omega-3 supplementation for migraine remain particularly sparse. The only prior pediatric trial, a crossover study of 27 adolescents, found no significant between-group difference in headache outcomes between fish oil and olive oil, though the small sample size may have limited statistical power to detect a clinically meaningful effect. Notably, that study did not measure erythrocyte omega-3 incorporation or circulating lipid mediators, leaving the biological response to supplementation in this population uncharacterized [18]. No prior pediatric randomized controlled trial has evaluated the biologic effects of omega-3 supplementation on lipid biomarker profiles in youth with migraine.

An important challenge in nutritional intervention research is determining whether supplementation produces a measurable change in circulating fatty acid status. This is compounded by the difficulty in identifying a control that is largely biologically inert within the context of the omega-3 physiologic pathway as well as accounting for concurrent dietary omega-6 PUFA intake [14]. The omega-3 PUFA index, defined as the combined percentage of EPA and DHA in erythrocytes or whole blood, is a validated biomarker of omega-3 fatty acid status associated with tissue incorporation in studies using erythrocyte-based measurement [19,20]; in the present study, whole-blood measurement via dried blood spot was used as a practical surrogate. This whole-blood measure is distinct from the conventional erythrocyte-based omega-3 index measured in isolated red blood cell membranes. Related lipid ratios, including the omega-6/omega-3 ratio and the arachidonic acid/EPA (AA/EPA) ratio, provide additional descriptive measures of circulating fatty acid profiles and relative precursor availability for downstream lipid mediator pathways [21,22,23]. Evaluation of these biomarkers may be especially valuable in pediatric pilot studies designed to establish biologic response and inform the development of future larger-scale clinical trials.

Therefore, the purpose of this pilot quasi-randomized clinical trial was to determine whether 12 weeks of omega-3 PUFA supplementation produces measurable changes in whole-blood EPA and DHA levels in children and adolescents with migraine, and to characterize changes in related lipid biomarker ratios and patient-reported clinical outcomes. We hypothesized that omega-3 supplementation would produce measurable shifts in lipid biomarker profiles compared with placebo.

## 2. Materials and Methods

### 2.1. Study Design and Participants

This study was a quasi-randomized, double-blind, placebo-controlled clinical trial evaluating the effects of omega-3 PUFA supplementation in children and adolescents with migraine. Participants were recruited from pediatric neurology clinics within the University of North Carolina (UNC) and Duke University Health Systems and assigned 1:1 using a sequential alternating allocation to receive either omega-3 PUFA supplementation or placebo daily for 12 weeks. Assessments were conducted at baseline and at the 12-week follow-up visit.

Eligible participants were children and adolescents between 10 and 17 years of age with a diagnosis of migraine based on the International Classification of Headache Disorders, 3rd edition (ICHD-3) [24] criteria, as confirmed by their current treating pediatric neurologist. Additional inclusion criteria included ability to provide assent and fluency in English. Exclusion criteria included known allergy to fish, seafood, or coconut oil; current use of omega-3 supplementation; and pregnancy or nursing status.

Recruitment and follow-up procedures were conducted between March 2025 and February 2026. Written informed consent was obtained from caregivers or legal guardians, and assent was obtained from participating children and adolescents prior to enrollment.

The sample size was determined to provide adequate statistical power for the primary biomarker outcome (change in omega-3 PUFA index). A power calculation indicated that a minimum of 21 participants per group would provide 80% power to detect a significant change in the omega-3 PUFA index (standardized effect size 0.865, two-sided alpha = 0.05). The assumed standardized effect size of 0.865 was based on prior omega-3 supplementation data demonstrating omega-3 index increases from ~4.9% to ~8.1% (Cohen’s d ≈ 1.88) across 14 published trials and represents a conservative estimate relative to the published literature [25]. A target enrollment of 80 participants (40 per group) was planned to account for anticipated attrition. Of 57 participants ultimately allocated, 44 (22 per group) completed the study, meeting the minimum sample size for the primary biologic outcome. Additional feasibility considerations informing the target enrollment, including recruitment yield, retention, and acceptability, will be reported in a companion feasibility manuscript.

The study protocol was approved by the University of North Carolina Institutional Review Board (IRB#24-2715, 26 February 2025) and conducted in accordance with the Declaration of Helsinki. The trial was registered at ClinicalTrials.gov (Identifier: NCT06899074; date of registration: 20 March 2025), where the full study protocol is publicly available, and is reported in accordance with the Consolidated Standards of Reporting Trials (CONSORT) guidelines [26]. The completed CONSORT checklist is provided as Supplementary Material.

### 2.2. Allocation and Blinding

Participants were assigned 1:1 using a sequential alternating allocation approach to receive either omega-3 PUFA supplementation or placebo to maintain balanced group enrollment throughout the study. Allocation assignments were determined sequentially at the time of enrollment. Minor imbalance in final group sizes (29 active intervention, 28 placebo) reflects the sequential nature of enrollment and participant discontinuation in this small pilot trial. Potentially eligible participants were screened and enrolled by trained research personnel. The omega-3 PUFA supplement was a commercially available strawberry-flavored formulation (Nordic Naturals Children’s DHA; Nordic Naturals, Watsonville, CA, USA); the coconut oil placebo was independently compounded with strawberry flavoring (Chapel Hill Compounding, Chapel Hill, NC, USA) to match. Both interventions were dispensed in identical sequentially labeled containers. Allocation assignments and dispensing of study intervention materials were managed by personnel not involved in outcome assessment. Participants, caregivers, investigators, and study personnel involved in data collection and outcome assessment remained blinded to treatment assignment until completion of the primary analyses.

### 2.3. Intervention

Participants assigned to the intervention group received a daily oral omega-3 PUFA supplement (Nordic Naturals, Watsonville, CA, USA) for 12 weeks. The liquid formulation contained EPA and DHA in a 40:60 ratio, providing 340 mg EPA and 510 mg DHA per teaspoon. Participants assigned to the placebo group received a matched daily liquid coconut oil preparation (Chapel Hill Compounding, Chapel Hill, NC, USA) for 12 weeks. Independent gas chromatography-mass spectrometry analysis demonstrated that the fractionated coconut oil consisted predominantly of caprylic acid (56.0%) and capric acid (43.3%), consistent with the expected medium-chain triglyceride profile.

Participants and caregivers were instructed to administer one teaspoon orally each day and to avoid initiating additional omega-3 supplementation during the study period. Participants were also asked not to substantially alter their usual dietary habits, omega-3 intake, or migraine management regimen unless medically necessary. Adherence and tolerability were monitored throughout the study period.

### 2.4. Dietary Assessment

Dietary intake was assessed for each participant using two 24-h dietary recalls (one weekday and one weekend day) collected using the Nutrition Data System for Research (NDSR; Nutrition Coordinating Center, University of Minnesota, Minneapolis, MN, USA). Dietary recalls were conducted at baseline and at the 12-week follow-up visit by trained staff from the UNC Nutrition and Obesity Research Center Clinical and Community Human Assessment and Intervention Core: Behavioral Assessment via telephone administration. For analysis, dietary intake variables were averaged across recalls within participant and study timepoint to account for day-to-day variability in intake.

### 2.5. Study Procedures and Outcome Measures

Study assessments were conducted at baseline and at the 12-week follow-up visit. At both time points, participants completed validated patient-reported outcome measures and provided a finger-prick blood sample for lipid biomarker analysis. Demographic information, headache history, and medication use were collected at baseline. Participants also completed dietary assessments at baseline and week 12 as described above.

Study adherence was assessed through structured telephone follow-up calls conducted at weeks 4 and 8. During these calls, caregivers were asked standardized questions regarding supplement administration, missed doses, tolerability, and any interval changes in medical history or medication use during the intervention period.

Feasibility outcomes including recruitment, retention, adherence, biospecimen completion, and intervention acceptability will be reported in a companion manuscript.

#### 2.5.1. Primary Outcome

The primary outcome was change in omega-3 PUFA index from baseline to 12 weeks. Omega-3 PUFA index was defined as the combined percentage of EPA and DHA relative to total identified fatty acids in whole blood. Blood samples were collected using a finger-prick dried blood spot method and analyzed by OmegaQuant Analytics (Sioux Falls, SD, USA) using the Omega-3 Index Plus panel, with fatty acid composition quantified by gas chromatography with flame ionization detection. The panel returned summary measures only: the omega-3 PUFA index (combined percentage of EPA and DHA), the omega-6/omega-3 ratio, and the AA/EPA ratio, each expressed relative to total fatty acids. Participant-level concentrations of the individual fatty acids composing these measures were not reported.

#### 2.5.2. Secondary and Exploratory Outcomes

Secondary outcomes included changes in patient-reported clinical outcomes from baseline to 12 weeks; the study was not powered to detect between-group differences in these measures. Change in lipid biomarker ratios from baseline to 12 weeks was an exploratory outcome, which included the omega-6/omega-3 ratio and AA/EPA ratio derived from whole blood fatty acid analysis.

Pain intensity and pain interference were assessed using the validated Patient-Reported Outcomes Measurement Information System (PROMIS) Pediatric Pain Intensity Measure [27] and PROMIS Pediatric Pain Interference Scale [28], respectively, both developed for children and adolescents ages 8–17 years. Higher scores indicate greater pain severity and pain-related interference.

Migraine-related disability was assessed using the validated Pediatric Migraine Disability Assessment (PedMIDAS) [29], a six-item questionnaire evaluating migraine-related disability over the previous three months. Total scores range from 0 to over 50, with higher scores indicating greater migraine-related disability.

Symptoms of anxiety and depression were assessed using the validated Revised Children’s Anxiety and Depression Scale short version (RCADS-25) [30], which generates standardized scores for internalizing symptoms in children and adolescents, with higher scores indicating greater psychological distress.

All questionnaires were completed electronically at baseline and at the 12-week follow-up visit.

Pediatric Quality of Life Inventory (PedsQL) Measurement Model [31], which was prespecified as a secondary outcome in the trial protocol and registered at ClinicalTrials.gov NCT06899074, intended to assess pediatric quality of life. This self-report measure was intended to be administered at baseline and Week 12 via the study’s electronic data capture system, Carolina Data Acquisition and Reporting Tool (CDART). During data cleaning and preparation for analysis, it was discovered that a programming error in the CDART system resulted in an incorrect self-report instrument being loaded in place of the PedsQL Measurement Model. Specifically, the PedsQL Pediatric Pain Questionnaire instrument was loaded into the CDART platform, resulting in systematic omission of all PedsQL Measurement Model items across all participants. Because the study already included a prespecified measure of pain intensity using PROMIS Pediatric Pain Intensity, the erroneously administered instrument captured a construct that was redundant with an existing outcome rather than the distinct construct originally intended. This error was identified during data quality review on 19 May 2026, after enrollment was complete. The decision to exclude PedsQL Pediatric Pain Questionnaire data from the present analysis was made by the study team prior to unblinding. This change from the prespecified analysis plan was not motivated by internal or external outcome data but rather by the inability to assess the intended construct and the redundancy of the collected data with an existing prespecified construct.

#### 2.5.3. Acceptability Measures

At the 12-week follow-up visit, caregivers completed a study-specific caregiver exit questionnaire assessing perceived changes in migraine symptoms, willingness to use non-pharmacologic approaches for migraine management, and overall study experience. Overall acceptability of study participation will be reported in the companion feasibility manuscript; caregiver-reported perceived clinical changes and perspectives on migraine management are reported here as context for interpreting patient-reported outcomes.

### 2.6. Statistical Analysis

Baseline demographic and clinical characteristics were summarized by treatment group. Continuous variables were summarized using mean and standard deviation (SD) or median and interquartile range (IQR), with ranges reported where appropriate based on distributional characteristics and sample size. Age, migraine disability, and ordinal pain measures were summarized using median (IQR) and range. Categorical variables, including sex at birth, race, and ethnicity, were summarized as counts and percentages. The omega-3 PUFA index, lipid ratios (omega-6/omega-3 and AA/EPA), and PROMIS pediatric pain measures (reported as T-scores [27]) were summarized using mean (SD). No formal statistical comparisons between treatment groups were performed for baseline characteristics. Analyses were conducted using complete-case data from participants who completed the 12-week follow-up assessment. A complete-case approach was selected given the pilot nature of the study and because the primary biologic outcome (omega-3 PUFA index) required paired baseline and 12-week biospecimen data for meaningful interpretation. Primary, secondary, and exploratory outcomes were assessed only at baseline and week 12, and no imputation procedures were performed for missing outcome data. Attrition rates, reasons for discontinuation, and characteristics of non-completers are reported to facilitate assessment of potential attrition bias (see Section 3.1).

The primary endpoint was the 12-week change in omega-3 PUFA index. Exploratory biochemical endpoints included change in the omega-6/omega-3 ratio and the AA/EPA ratio. Between-group differences in primary and exploratory biochemical endpoints were evaluated using analysis of covariance (ANCOVA), with treatment group as the independent variable and the corresponding baseline value included as a covariate.

Secondary patient-reported outcomes, including PROMIS Pediatric Pain Intensity, PROMIS Pediatric Pain Interference, PedMIDAS, and the RCADS-25, were analyzed using analogous ANCOVA models, with the 12-week value as the dependent variable and adjustment for baseline values.

Responses from the caregiver exit questionnaire were summarized descriptively using frequencies and percentages. Exploratory descriptive analyses and data visualizations were also conducted to characterize individual variability and longitudinal outcome trajectories across treatment groups.

This approach was used to improve precision and account for baseline variability and potential regression to the mean. Model assumptions, including linearity and homogeneity of regression slopes, were assessed and met. Results are reported as adjusted mean differences with 95% confidence intervals (CIs) and two-sided *p*-values. All analyses were conducted using Stata version 19 SE (StataCorp, College Station, TX, USA).

Because the PedsQL Measurement Model data were not collected due to the administration of an incorrect instrument, no statistical analysis of this outcome was performed. The erroneously collected PedsQL Pediatric Pain Questionnaire data were not substituted for the prespecified outcome, as this would constitute a post hoc change to the outcome measurement instrument and would not assess the intended construct of pediatric quality of life. Furthermore, reporting the erroneously collected data as a secondary measure of pain intensity was judged to be inappropriate given the redundancy with the prespecified PROMIS Pediatric Pain Intensity and the risk of outcome multiplicity without a priori justification. All other prespecified outcomes were analyzed as planned.

## 3. Results

### 3.1. Participant Flow and Retention

**Figure 1:**
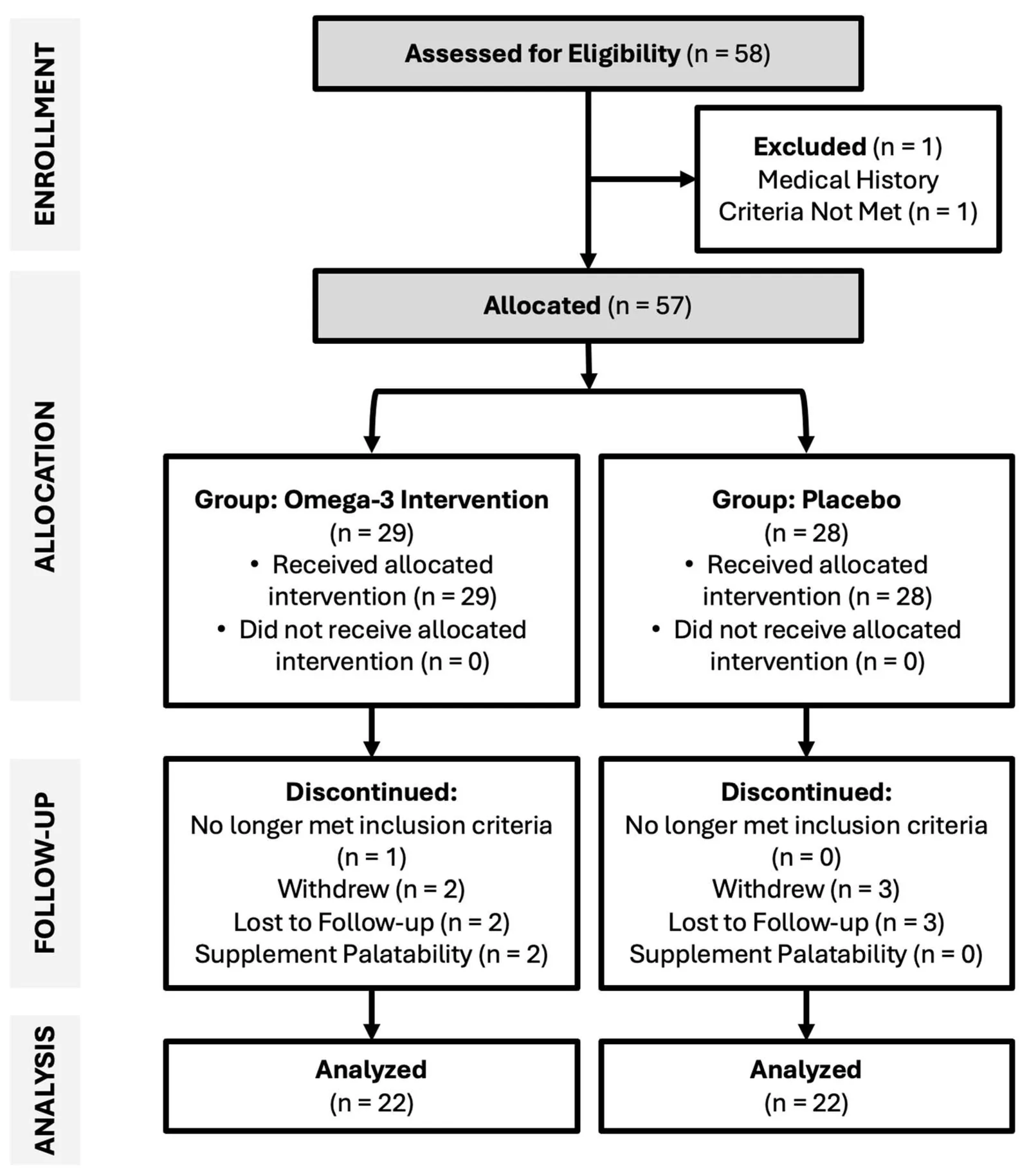
CONSORT flow diagram of participant enrollment, allocation, follow-up, and analysis. A total of 58 individuals were assessed for eligibility, of whom 57 were allocated to the omega-3 PUFA intervention group (*n* = 29) or placebo group (*n* = 28). Of the allocated participants, 44 (22 per group) completed the 12-week follow-up visit and were included in the final analysis.

Participant flow through the study is shown in Figure 1. A total of 58 individuals were assessed for eligibility, of whom 57 were allocated to the omega-3 PUFA intervention group (*n* = 29) or placebo group (*n* = 28). Thirteen participants discontinued after initiating the intervention: five withdrew from study participation, five were lost to follow-up, two discontinued due to difficulties related to supplement palatability or taste, and one no longer met inclusion criteria. Attrition was somewhat higher in the intervention group (7 of 29, 24.1%) compared with the placebo group (6 of 28, 21.4%), though the study was not designed to formally compare discontinuation rates.

Of the allocated participants, 44 (77.2%) completed the 12-week follow-up visit and were included in the final analysis (22 participants per group), corresponding to an overall attrition rate of 22.8%. Participants who completed the study also completed patient-reported outcome measures, dietary assessments, and lipid biomarker collection at the 12-week follow-up visit. No serious adverse events related to study participation were reported.

### 3.2. Baseline Participant Characteristics

Baseline demographic and clinical characteristics are summarized in Table 1. The median age was 14 years in both groups, and the distribution of sex at birth was similar between treatment arms. Participants were predominantly White and non-Hispanic/non-Latino, with comparable racial and ethnic distributions across groups. Similar baseline demographics were noted for the 13 non-completers, with 9 being female (69.2%), 2 being male (15.4%), 2 preferring not to disclose gender (15.4%), and 9 (69.2%) being White and/or non-Hispanic/non-Latino.

Baseline lipid biomarker profiles were also generally similar between groups. Mean omega-3 PUFA index values were 4.12 (SD 0.61) in the placebo group and 3.83 (SD 0.62) in the intervention group. Mean omega-6/omega-3 ratios and AA/EPA ratios were comparable between treatment arms.

Baseline patient-reported outcomes demonstrated relatively modest overall symptom burden. PROMIS Pediatric Pain Intensity T-scores were within the normal range in both groups, while PROMIS Pediatric Pain Interference scores were mildly elevated. Median PedMIDAS scores reflected variable migraine-related disability across participants. Baseline psychological distress scores were similar between groups.

Mean dietary omega-3 fatty acid intake at baseline was 1.56 (SD 1.07) g/day in the placebo group and 1.70 (SD 1.41) g/day in the intervention group, with dietary EPA + DHA intake very low in both groups (approximately 0.12 g/day), consistent with typical U.S. pediatric intake patterns [32] (Supplementary Table S1).

### 3.3. Primary Outcome: Omega-3 PUFA Index

At 12 weeks, participants receiving omega-3 PUFA supplementation (*n* = 22) demonstrated a significantly greater increase in omega-3 PUFA index compared with placebo (Table 2). In the ANCOVA model adjusting for baseline omega-3 PUFA index values, the adjusted mean difference between groups was 1.64 (95% CI, 0.94 to 2.35; *p* < 0.001).

Mean omega-3 PUFA index values increased from baseline in the intervention group (within-group mean difference of 1.67%, SE 0.32), consistent with biologic response to supplementation, whereas values remained relatively stable in the placebo group (within-group mean difference of 0.02%, SE 0.10). These findings indicate successful alteration of circulating whole-blood omega-3 fatty acid levels following the 12-week intervention period.

### 3.4. Exploratory Lipid Biomarker Outcomes

Changes in exploratory lipid biomarker outcomes also favored the intervention group (Table 3). In adjusted analyses, participants receiving omega-3 PUFA supplementation (*n* = 22) demonstrated greater reductions in the omega-6/omega-3 ratio compared with placebo (adjusted mean difference, −2.57; 95% CI, −4.25 to −0.90; *p* = 0.003).

Similarly, the intervention group demonstrated a significantly greater reduction in the AA/EPA ratio relative to placebo (adjusted mean difference, −13.52; 95% CI, −21.55 to −5.49; *p* = 0.002).

Overall, these findings demonstrate consistent shifts in lipid biomarker profiles following omega-3 supplementation over the 12-week intervention period. These ratio changes are mathematically related to the primary outcome, as they are driven by the increase in omega-3 fatty acids in the denominator rather than reductions in omega-6 levels, and therefore do not represent independent biological findings. They are reported as descriptive measures of relative fatty acid precursor availability.

### 3.5. Secondary Patient-Reported Outcomes

Changes in patient-reported outcomes at 12 weeks are summarized in Table 4. No significant between-group differences were observed for PROMIS Pediatric Pain Intensity, PROMIS Pediatric Pain Interference, migraine-related disability, or psychological distress following the intervention period.

**Figure 2:**
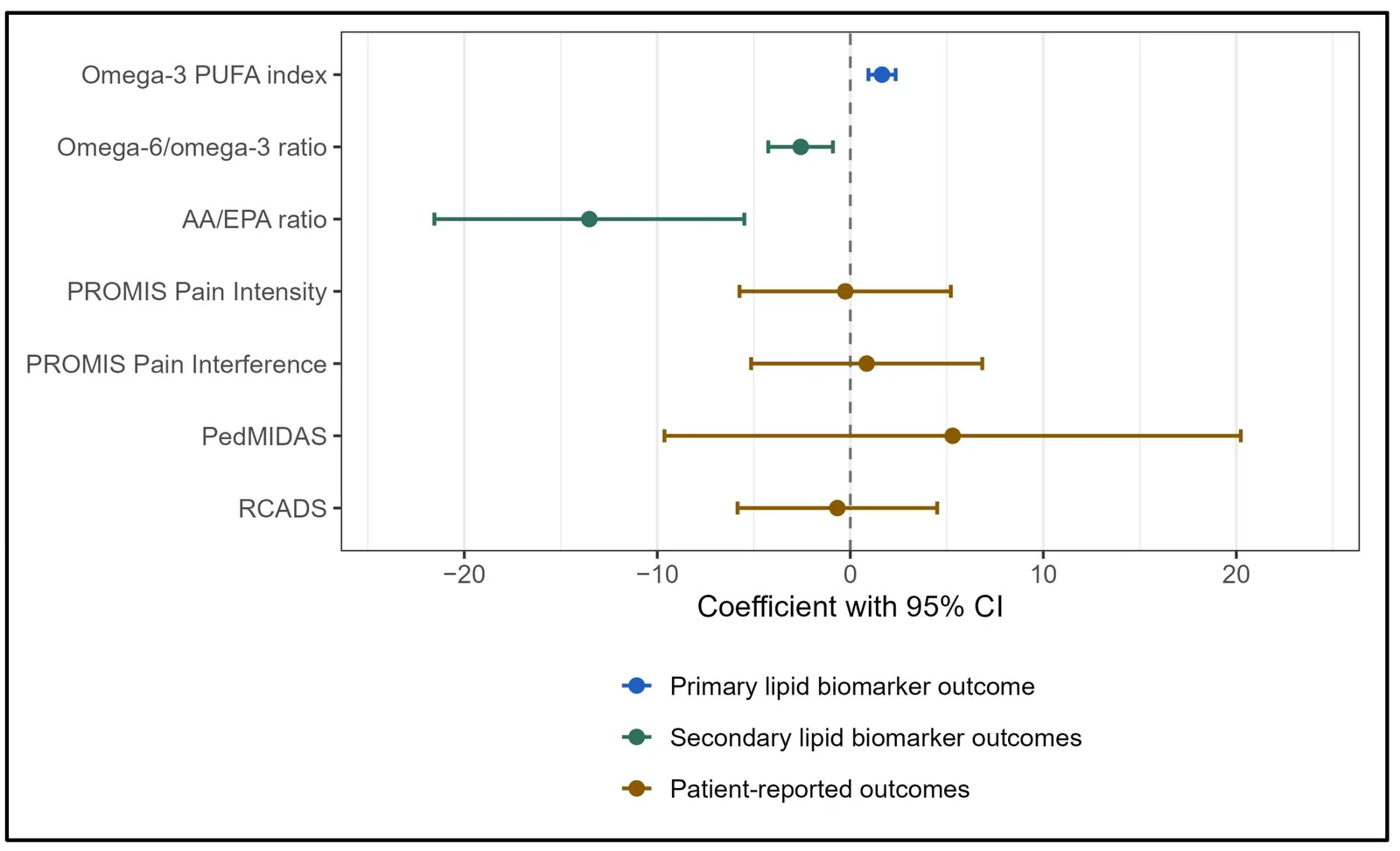
Adjusted mean differences and 95% confidence intervals for 12-week changes in lipid biomarker and patient-reported outcomes between the omega-3 PUFA intervention (*n* = 22) and placebo groups (*n* = 22). Positive and negative values reflect the direction of change for each outcome measure. Lipid biomarker outcomes demonstrated significant between-group differences favoring omega-3 supplementation; ratio-based endpoints share a mathematical dependency with the primary omega-3 PUFA index outcome. Patient-reported outcomes showed substantial variability with no statistically significant between-group differences.

In adjusted analyses, the estimated between-group difference in change for PROMIS Pediatric Pain Intensity was −0.26 (95% CI, −5.74 to 5.21; *p* = 0.923), and the estimated between-group difference for PROMIS Pediatric Pain Interference was 0.85 (95% CI, −5.14 to 6.84; *p* = 0.776). Similarly, no significant between-group differences were observed for PedMIDAS scores (adjusted mean difference, 5.30; 95% CI, −9.63 to 20.23; *p* = 0.478) or RCADS-25 scores (adjusted mean difference, −0.67; 95% CI, −5.84 to 4.50; *p* = 0.795). A visual summary of adjusted mean differences and 95% confidence intervals across lipid biomarker and patient-reported outcomes is presented in Figure 2. Individual longitudinal trajectory plots illustrating within-person variability across study outcomes are provided in Supplementary Figure S1.

### 3.6. Adherence Data

Adherence to the study intervention was assessed via caregiver-reported missed days at Week 4 and Week 8 adherence calls. Caregiver-reported adherence was high across both groups; detailed adherence data will be reported in the companion feasibility manuscript. The significant increase in omega-3 PUFA index observed in the intervention group provides objective biomarker confirmation of supplement intake consistent with caregiver-reported adherence.

### 3.7. Caregiver Exit Questionnaire Findings

Caregiver-reported acceptability of daily supplement administration and openness to future dietary supplement use were high across both groups; detailed acceptability findings will be reported in the companion feasibility manuscript. Regarding observed changes during the study period, 61.4% of caregivers reported fewer or less severe migraines in their child, 29.5% reported improved emotional well-being, and 25.0% reported improved participation in daily activities. Additionally, 65.9% of caregivers reported increased interest in non-pharmacologic migraine management approaches after study participation. Caregiver exit questionnaire findings related to caregiver perspectives on perceived treatment benefits are provided in Table 5.

### 3.8. Dietary Recall Findings

Descriptive dietary intake data showed no systematic between-group differences in mean energy, total fat, or omega-3 and omega-6 fatty acid intake from baseline to Week 12, though substantial individual variability was observed (Supplementary Table S1). These data provide limited reassurance that the observed biomarker changes were not driven by major shifts in habitual dietary intake, though the precision of this assessment is constrained by the small sample size and inherent variability of 24-hour recall methods.

## 4. Discussion

In this quasi-randomized clinical trial of 44 children and adolescents with migraine defined according to ICHD-3 criteria, supplementation with marine-derived omega-3 PUFAs produced robust and consistent changes in the primary outcome, which was circulating lipid biomarkers, but did not result in measurable improvements in the exploratory outcome of pain or related patient-reported outcomes over 12 weeks. Specifically, the active intervention significantly increased the omega-3 PUFA index and reduced both the omega-6/omega-3 ratio and the AA/EPA ratio, indicating a measurable change in whole-blood fatty acid composition. Dietary recall data demonstrated relatively stable energy intake, total fat intake, and omega-3 and omega-6 fatty acid intake across the 12-week study period in both groups, suggesting that the observed biomarker changes were primarily attributable to the study intervention rather than substantial changes in habitual dietary intake. Because the exploratory ratio endpoints share a mathematical dependency with the primary outcome (i.e., both the omega-6/omega-3 and AA/EPA ratios are driven by the same omega-3 denominator) these findings should be interpreted as complementary descriptive measures rather than independent evidence of a distinct biomarker response. In contrast, no between-group differences were observed across validated measures of pain intensity, pain interference, migraine-related disability, or psychological distress.

While increases in blood EPA and DHA following omega-3 supplementation are well-documented in adult populations, the present findings extend this evidence in several important ways. This is the first quasi-randomized controlled trial to measure omega-3 PUFA index response in children and adolescents with migraine, a population in which the response magnitude was unknown. The observed data also provide dose–response information essential for planning future adequately powered efficacy trials, including whether 850 mg/day EPA + DHA is sufficient to reach proposed target omega-3 index ranges in this age group.

The observed increases in whole-blood EPA and DHA are relevant to migraine pathophysiology because these fatty acids serve as precursors to downstream lipid mediators that modulate nociceptive and neuroinflammatory signaling. EPA-derived resolvins (and DHA-derived resolvins, protectins, and maresins suppress pro-inflammatory cytokines, modulate transient receptor potential channels on trigeminal nociceptors, and actively promote resolution of neuroinflammation [33,34]. Notably, Zamora et al. (2026) recently demonstrated that blood EPA levels are significantly associated with EPA concentrations in cranial arteries, meninges, and trigeminal ganglia, supporting the use of blood fatty acid measurements as a proxy for tissue-level changes in structures directly involved in headache pathogenesis [35]. In the Ramsden et al. (2021) trial, dietary increases in EPA and DHA altered circulating nociceptive oxylipin derivatives, including the antinociceptive mediator 17-hydroxydocosahexaenoic acid, although classic headache mediators such as calcitonin gene-related peptide and prostaglandin E2 were not significantly altered [15]. The present study measured upstream precursor fatty acid availability only and did not assess downstream lipid mediators, inflammatory cytokines, or SPMs directly. Whether the magnitude of whole-blood EPA and DHA increase observed here is sufficient to meaningfully alter these downstream pathways in youth with migraine remains an important question for future investigation.

The relative contributions of EPA and DHA to brain-level effects merit specific consideration, particularly because the study formulation was DHA-predominant (340 mg EPA and 510 mg DHA per daily dose). Although EPA and DHA share many neuroactive properties, the available evidence suggests partially distinct roles. EPA has been more consistently associated with antidepressant and anti-inflammatory effects, with higher-dose EPA (>1 g/day) improving depressive symptoms in inflammatory and pediatric or adolescent populations, an effect linked to increases in EPA-derived pro-resolving lipid mediators [36,37]. In an adult episodic-migraine trial, high-dose EPA (1.8 g/day) reduced migraine days and concurrently improved anxiety, depression, and quality of life [16]. DHA, by contrast, is the predominant structural omega-3 fatty acid in neuronal membranes and appears at least as effective as EPA in modulating glial activation and suppressing pro-inflammatory cytokines such as TNF-α, IL-1β and IL-6 [38]. Both fatty acids serve as substrates for specialized pro-resolving mediators that shift lipid signaling from a pro-inflammatory to a pro-resolving state and dampen microglial activation and trigeminal nociception relevant to migraine pathophysiology [7]. These distinctions are relevant to the interpretation of the present findings: a relatively low-dose, DHA-predominant formulation may be less optimal for the mood- and inflammation-related effects that have been most consistently linked to EPA, and dosing ratio meta-analyses indicate that EPA:DHA ratios below 1.0 favor cytokine reduction whereas higher ratios more effectively raise blood EPA and lower arachidonic acid [21]. This consideration may help contextualize the absence of between-group differences in patient-reported pain and psychological distress outcomes and should inform formulation and dosing decisions in future adequately powered efficacy trials in youth with migraine.

These biomarker findings are consistent with prior adult omega-3 intervention trials in migraine [16,18]. Most notably, in the Ramsden et al. three-arm randomized trial, dietary interventions increasing EPA and DHA significantly altered circulating omega-3 and omega-6 fatty acids and their nociceptive oxylipin derivatives, with concurrent reductions in headache frequency and severity over 16 weeks [15]. The present study extends these findings by demonstrating that omega-3 supplementation can produce measurable shifts in lipid biomarker profiles in a pediatric migraine population, a population in which randomized trial data on omega-3 fatty acids remain extremely limited, where shorter RBC lifespan relative to adults may allow the omega-3 index to reach steady state more rapidly, and where lower age-typical baseline values may facilitate a proportionally larger response at a given dose [20,25,39]. Moreover, the magnitude of omega-3 index response to supplementation is not fully predictable even in adults: the largest modeling study to date found that dose, baseline status, and formulation type explained only 62% of response variance, leaving 38% unexplained [25]. In pediatric populations specifically, body weight has been shown to significantly modify omega-3 accumulation following supplementation [40], further highlighting why the biologic response in youth with migraine could not be assumed from existing adult data. Because the dried blood spot method measures EPA plus DHA in whole blood rather than isolated erythrocyte membranes, absolute values are not directly interchangeable with the conventional erythrocyte-based omega-3 index without conversion algorithms developed primarily in adult populations. However, this method is a validated surrogate in adults and the within-subject change design supports valid assessment of the intervention’s biological effect, while underscoring the need for pediatric-specific reference ranges and DBS validation studies in this age group [41,42].

The absence of detectable clinical effects in the present study may reflect limited statistical power inherent to this pilot sample, an insufficient dose or intervention duration, or a true absence of clinical benefit at this dose in this population. These explanations are not mutually exclusive, and the present study was not designed to distinguish among them. With a small sample size and substantial variability in pediatric pain outcomes, the present study was not powered to detect modest but potentially clinically meaningful differences, and the confidence intervals for several outcomes remain wide. In addition, a 12-week intervention period may be insufficient for lipid-mediated biological changes to translate into downstream modulation of neuroinflammatory and pain processing pathways relevant to pediatric migraine. Notably, the Ramsden et al. trial used a 16-week intervention period and provided foods accounting for two-thirds of daily energy intake, a substantially more intensive dietary manipulation than supplementation alone, and still did not achieve significant improvement on the HIT-6 quality-of-life measure despite significant reductions in headache frequency [15]. Baseline symptom levels in the present cohort were relatively modest and heterogeneous (e.g., PROMIS Pediatric Pain Intensity T-scores were within the normal range in both groups and PedMIDAS scores varied widely [range 0–180]), further reducing sensitivity to detect change in a small sample.

A high placebo response is well-recognized in pediatric migraine trials, with rates nearly twice as high as those in adults [43] and exceeding 60% in the landmark CHAMP trial [5]. The American Academy of Neurology has noted that this robust placebo response likely has a biological basis and that trial designs associated with lower placebo response rates include crossover designs, single-center studies, and small sample sizes [4]. Although the present study was not designed to detect clinical efficacy, the high placebo response characteristic of pediatric migraine trials highlights the challenge of demonstrating between-group differences in patient-reported outcomes in this population and should inform the design of future larger-scale trials.

Several study design considerations merit discussion. Coconut oil was selected as the placebo because it is composed predominantly of medium-chain triglycerides (caprylic acid 56.0%, capric acid 43.3%) that are rapidly absorbed and oxidized for energy rather than incorporated into cell membranes or metabolized through eicosanoid or docosanoid pathways implicated in migraine pathophysiology [44,45], unlike olive oil or corn oil placebos which may exert immunomodulatory effects that obscure between-group differences. The omega-6/omega-3 ratio and AA/EPA ratio were included as exploratory biomarkers to characterize the broader lipid profile response, while the omega-3 PUFA index served as the primary outcome. As noted above, these ratio changes were driven by increases in omega-3 levels rather than decreases in omega-6 levels and do not independently demonstrate anti-inflammatory effects; future studies should measure downstream lipid mediators to characterize biologic effects more precisely.

The baseline omega-3 PUFA index values observed in this cohort (mean ~4%) are consistent with population-level data indicating that most U.S. children have omega-3 levels well below the target range of 8–11% proposed for optimal health [19,32]. These data suggest that the pediatric migraine population studied here had low baseline omega-3 status typical of Western youth, and that the 12-week supplementation period, while producing statistically significant increases, did not raise the omega-3 index into the proposed target range in the majority of participants.

This study has several strengths. It is among the first quasi-randomized, double-blind, placebo-controlled trials to evaluate the biologic effects of omega-3 PUFA supplementation in children and adolescents with migraine. The use of validated whole-blood fatty acid endpoints (omega-3 PUFA index, omega-6/omega-3 ratio, AA/EPA ratio) provided objective measures of whole-blood omega-3 status, consistent with recommendations from the International Society for the Study of Fatty Acids and Lipids that omega-3 levels should be measured at baseline and post-intervention in supplementation trials to confirm biologic response [46]. The inclusion of validated patient-reported outcome measures (PROMIS, PedMIDAS, RCADS-25) and dietary assessment via 24-h recalls further strengthens the study design.

Limitations and Future Directions. Several limitations should be acknowledged. First, treatment was assigned by sequential alternating allocation rather than concealed computer-generated randomization; this quasi-randomized approach is potentially predictable and does not eliminate selection bias, although baseline characteristics were similar between groups (Table 1). Future trials should use concealed computer-generated randomization. Second, of 57 allocated participants, 44 (77.2%) completed the study (overall attrition 22.8%; 24.1% intervention vs. 21.4% placebo), with supplement palatability cited among reasons for discontinuation, consistent with retention challenges reported in pediatric omega-3 supplementation trials [47]. Primary analyses were complete-case because the biomarker endpoint required paired baseline and 12-week specimens; combined with this attrition, this introduces a risk of attrition bias affecting the effect estimates and their generalizability. Future trials should pre-specify intention-to-treat analyses with multiple imputation or sensitivity analyses and adopt retention strategies and more palatable formulations. Third, as a small pilot, this study was not powered to detect between-group differences in patient-reported outcomes, and confidence intervals were wide. The absence of significant clinical effects should not be read as evidence of no benefit, nor attributed solely to limited power, as insufficient dose, insufficient duration, or a genuine absence of effect at this dose remain non-mutually exclusive explanations. The small sample also precluded evaluation of whether baseline omega-3 status modified response magnitude; future larger trials should examine this potential effect modifier. Fourth, measurement was limited. Whole-blood EPA + DHA was quantified from dried blood spots rather than isolated erythrocyte membranes, so values are not directly interchangeable with the conventional erythrocyte-based omega-3 index and membrane-specific incorporation was not demonstrated. The Omega-3 Index Plus panel used here returned only summary measures (the omega-3 PUFA index, the omega-6/omega-3 ratio, and the AA/EPA ratio) and did not provide participant-level concentrations of the individual constituent fatty acids (e.g., EPA, DHA, arachidonic acid, linoleic acid, total omega-3, total omega-6). As a result, a full fatty acid profile could not be tabulated, and the extent to which the observed ratio changes were driven by rising omega-3 versus stable omega-6 could only be inferred rather than shown directly at the component level. Downstream lipid mediators, cytokines, and specialized pro-resolving mediators were not measured, and headache frequency was not captured with a prospective daily diary, the preferred and most clinically relevant endpoint in migraine prevention trials [15,16]. A prespecified quality-of-life instrument (PedsQL Measurement Model) could not be reported owing to an electronic-data-capture error identified after enrollment; substitution of the erroneously collected pain-intensity data was judged inappropriate given the lack of pre-specification and risk of post hoc multiplicity [46], and the decision was made prior to unblinding. Fifth, the fixed 850 mg/day EPA + DHA dose over 12 weeks did not raise levels into the proposed 8–11% target range in most participants [19], and may have been insufficient to produce clinical effects, particularly in older adolescents approaching adult body weight; successful adult trials have used 1500–2000 mg/day [15,16,48]. The population was predominantly White and non-Hispanic, limiting generalizability, and caregiver-reported adherence may overestimate intake and did not capture the final four weeks.

Collectively, these limitations define the priorities for a definitive trial: concealed randomization, a larger and more demographically diverse cohort powered for clinical endpoints with pre-specified intention-to-treat analysis, higher or biomarker-guided dosing to a predefined target, prospective headache-diary capture of migraine frequency, and direct measurement of downstream lipid mediators to test whether whole-blood fatty acid changes translate into anti-inflammatory or antinociceptive effects. Future trials should also incorporate direct assessment of mood and central mechanisms, using validated affective outcome measures and neuroimaging (e.g., magnetic resonance imaging), to determine whether measurable whole-blood EPA and DHA changes translate into brain-level effects relevant to migraine and psychological distress, particularly in light of the differential neuromodulatory roles of EPA and DHA [36,49].

In conclusion, this trial showed that omega-3 PUFA supplementation produces measurable and consistent shifts in lipid biomarker profiles in children and adolescents with migraine, supporting a measurable whole-blood EPA + DHA response. These findings provide a foundation for future larger-scale trials with longer intervention duration, prospective headache diary assessment, and sufficient statistical power to evaluate whether omega-3 supplementation can reduce migraine frequency and disability in pediatric populations.

## 5. Conclusions

This quasi-randomized, double-blind, placebo-controlled trial showed that 12 weeks of omega-3 PUFA supplementation significantly increased the omega-3 PUFA index with corresponding reductions in the omega-6/omega-3 and AA/EPA ratios driven primarily by increased omega-3 levels, supporting a measurable whole-blood EPA + DHA response in children and adolescents with migraine. Adequately powered definitive trials are needed to determine the efficacy of omega-3 PUFA supplementation in adolescents suffering from migraine. Consistent whole-blood EPA + DHA responses support the rationale for future efficacy trials of omega-3 supplementation in this population.

## Figures and Tables

**Table 1:** Comparison of baseline characteristics in the two treatment groups.

	Placebo (*n* = 22)	Active Intervention (*n* = 22)
Demographic characteristics		
Age, median (IQR) [range], y	14 (11–16) [10–17]	14 (12–15) [11–16]
Sex at birth, *n* (%)		
Female	14 (64)	12 (55)
Male	8 (36)	10 (45)
Race, *n* (%)		
White	16 (73)	18 (82)
Black/African American	2 (9)	2 (9)
Other	4 (18)	0 (0)
Unreported	0 (0)	2 (9)
Ethnicity, *n* (%)		
Non-Hispanic/Non-Latino	20 (91)	17 (77)
Hispanic/Latino	1 (5)	2 (9)
Unknown/not reported	1 (5)	3 (14)
Biochemical characteristics		
Omega-3 PUFA index, mean (SD) ^(a)^	4.12 (0.61)	3.83 (0.62)
Omega-6/omega-3 ratio, mean (SD) ^(b)^	10.32 (2.52)	9.92 (2.03)
AA/EPA ratio, mean (SD) ^(b)^	38.60 (22.46)	36.41 (16.10)
Patient-reported outcomes		
Pediatric pain intensity, mean (SD) T-score	48.00 (10.19)	42.80 (6.65)
Pediatric pain interference, mean (SD) T-score	59.42 (6.65)	57.61 (5.69)
Migraine disability, median (IQR) [range]	27 (13–45) [0–180]	18 (6–72) [1–165]
Psychological distress, mean (SD)	53.32 (16.93)	50.24 (12.65)
Current pain, median (IQR) [range]	0 (0–1) [0–4]	0 (0–1) [0–2]
Worst pain in 7 days, median (IQR) [range]	2 (2–3) [0–4]	2 (1–2) [0–3]

Continuous variables are summarized as mean (SD) or median (IQR) based on distributional characteristics; no formal statistical comparisons were performed at baseline. ^(a)^ Omega-3 index is defined as the sum of eicosapentaenoic acid (EPA) and docosahexaenoic acid (DHA), expressed as a percentage of total whole blood fatty acids. ^(b)^ Lipid ratios were calculated at the individual level from measured fatty acid concentrations and are expressed as continuous ratios (e.g., omega-6/omega-3 = 6.0 indicates 6 units of omega-6 per 1 unit of omega-3).

**Table 2:** ANCOVA of 12-week change in the primary lipid biomarker endpoint.

Primary Endpoint	Adjusted Mean Difference ^(a)^	95% CI	*p*-Value
Change in omega-3 PUFA index	1.64	0.94 to 2.35	<0.001

^(a)^ The adjusted mean difference represents the adjusted mean difference between treatment groups in 12-week change in omega-3 PUFA index, adjusting for baseline values.

**Table 3:** ANCOVA of 12-week change in exploratory lipid biomarker endpoints.

Exploratory Biochemical Endpoints	Adjusted Mean Difference ^(a)^	95% CI	*p*-Value
Change in omega-6/omega-3 ratio	−2.57	−4.25 to −0.90	0.003
Change in AA/EPA ratio	−13.52	−21.55 to −5.49	0.002

^(a)^ The adjusted mean difference represents the adjusted mean difference between treatment groups in 12-week change in omega-6/omega-3 ratio and AA/EPA ratio, adjusting for baseline values.

**Table 4:** Adjusted mean difference in 12-week change in secondary endpoints.

Secondary Endpoints	Adjusted Mean Difference ^(a)^	95% CI	*p*-Value
Change in PROMIS Pediatric Pain Intensity Measure	−0.26	−5.74 to 5.21	0.923
Change in PROMIS Pediatric Pain Interference Measure	0.85	−5.14 to 6.84	0.776
Change in Pediatric Migraine Disability Assessment	5.30	−9.63 to 20.23	0.478
Change in Revised Children’s Anxiety and Depression Scale	−0.67	−5.84 to 4.50	0.795

^(a)^ Adjusted mean differences represent the estimated difference in 12-week change in secondary endpoints between the intervention and placebo groups, adjusting for baseline values.

**Table 5:** Caregiver-Reported Benefits Observed During Study Participation.

	Fewer or Less Severe Migraines	Improved Emotional Well-Being	Better Ability to Participate in Daily Activities	Improved Quality of Life for Our Family	Other	No Change
Total (*N* = 44)	27 (61.4%)	13 (29.5%)	11 (25.0%)	8 (18.2%)	5 (11.4%)	7 (15.9%)
Placebo (*n* = 22)	13 (59.1%)	5 (22.7%)	6 (27.3%)	3 (13.6%)	2 (9.1%)	3 (13.6%)
Intervention (*n* = 22)	14 (63.6%)	8 (36.4%)	5 (22.7%)	5 (22.7%)	3 (13.6%)	4 (18.2%)

Data are presented as the number and percentage of respondents in each category for the total sample (*N* = 44) and by group (Placebo, *n* = 22; Intervention, *n* = 22). Respondents could select multiple options; therefore, percentages exceed 100%.

## Data Availability

The original contributions presented in this study are included in the article/Supplementary Materials. Further inquiries can be directed to the corresponding author.

## Supplementary Materials

The following supporting information can be downloaded at: https://www.mdpi.com/article/10.3390/nu18182983/s1, Checklist S1: CONSORT 2010 Checklist of Information to Include When Reporting a Pilot or Feasibility Trial [26]. Figure S1: Individual longitudinal trajectories and distributions of baseline and week 12 outcome measures by treatment group. Plots illustrate within-person variability and heterogeneity of response across lipid biomarker and patient-reported outcome measures among participants receiving omega-3 PUFA supplementation or placebo. Table S1. Dietary Intake Assessed via the Nutrition Data System for Research: Mean Values ± SD at Baseline and Post-Intervention.

## Abbreviations

The following abbreviations are used in this manuscript:

AA	arachidonic acid
ANCOVA	Analysis of Covariance
CDART	Carolina Data Acquisition and Reporting Tool
CI	confidence intervals
CONSORT	Consolidated Standards of Reporting Trials
DHA	docosahexaenoic acid
EPA	eicosapentaenoic acid
ICHD-3	International Classification of Headache Disorders, 3rd edition
IL	interleukin
IQR	interquartile range
IRB	Institutional Review Board
NC TraCS	NC Translational and Clinical Sciences Institute
NCATS	National Center for Advancing Translational Sciences
NDSR	Nutrition Data System for Research
PedMIDAS	Pediatric Migraine Disability Assessment
PedsQL	Pediatric Quality of Life Inventory
PROMIS	Patient-Reported Outcomes Measurement Information System
PUFA	polyunsaturated fatty acids
RCADS-25	Revised Children’s Anxiety and Depression Scale short version
SD	standard deviation
SPM	specialized pro-resolving mediator
