# Supplementary material for: Omega-3 Supplementation Modulates Whole-Blood EPA and DHA Without Improving Patient-Reported Outcomes in Youth with Migraine: A Quasi-Randomized Pilot Trial
Source: Nutrients. 2026 Sep 11;18(18):2983. doi: 10.3390/nu18182983 (PMC13610828; doi:10.3390/nu18182983)
Supplement: Supplementary file 1 [file nutrients-18-02983-s001.zip › Supplementary Figure S1 v2.pdf]

nutrients

Supplementary Figure S1.

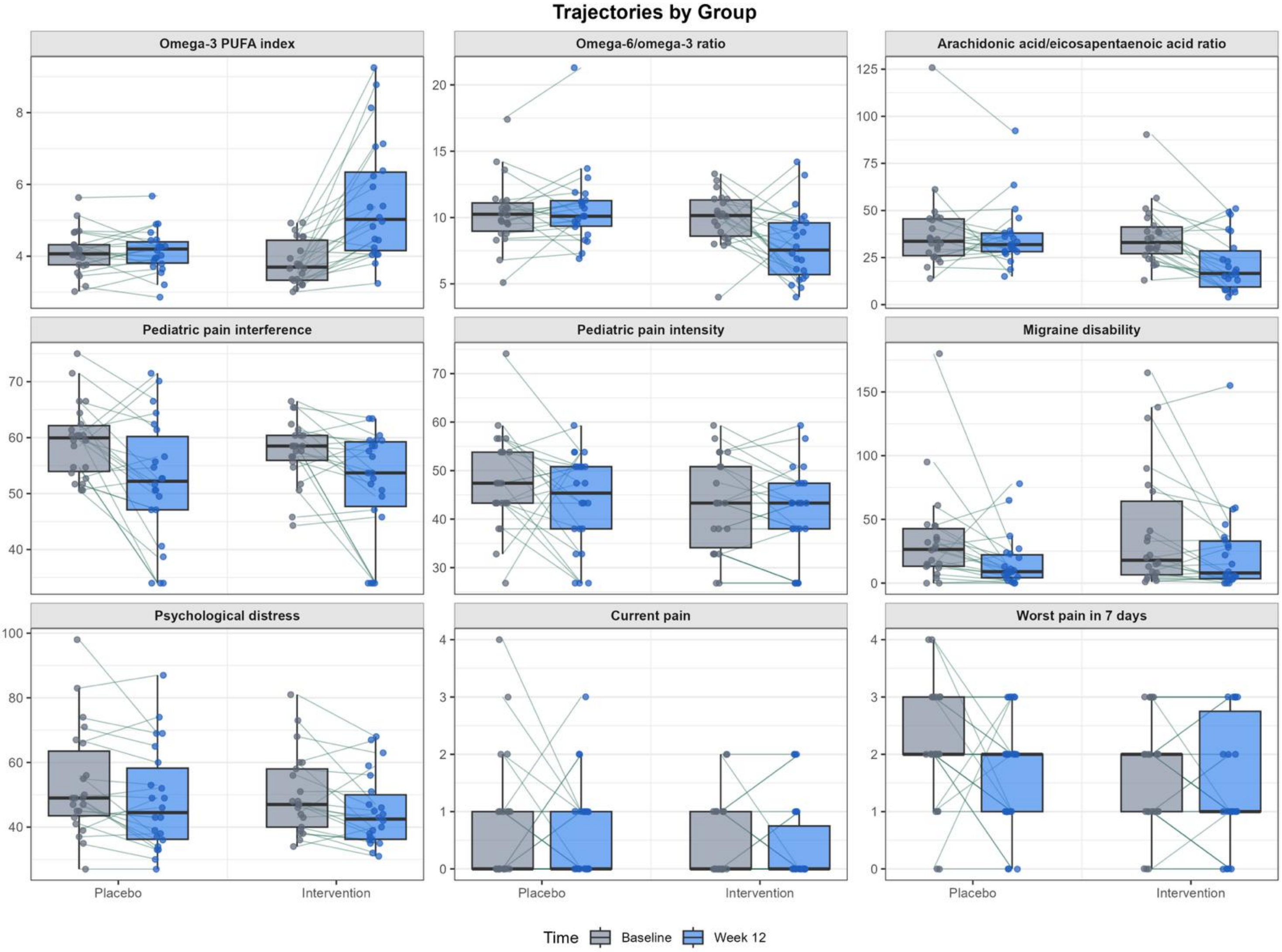

Supplementary Figure S1. Individual longitudinal trajectories and distributions of baseline and week 12 outcome measures by treatment group. Plots illustrate within-person variability and heterogeneity of response across lipid biomarker and patient-reported outcome measures among participants receiving omega-3 PUFA supplementation or placebo.
