# Supplementary material for: Omega-3 Supplementation Modulates Whole-Blood EPA and DHA Without Improving Patient-Reported Outcomes in Youth with Migraine: A Quasi-Randomized Pilot Trial
Source: Nutrients. 2026 Sep 11;18(18):2983. doi: 10.3390/nu18182983 (PMC13610828; doi:10.3390/nu18182983)
Supplement: Supplementary file 1 [file nutrients-18-02983-s001.zip › Supplementary Table S1 062926.pdf]

nutrients

MDPI

Table S1. Dietary Intake Assessed via the Nutrition Data System for Research: Mean Values ± SD at Baseline and Post-Intervention

	Intervention (n = 22)		Placebo (n = 22)	
	**Baseline**	**Week 12**	**Baseline**	**Week 12**
Total weight of food (g)	2480.07 ± 1277.40	2212.17 ± 1066.44	2436.82 ± 1059.28	2128.63 ± 893.24
Energy (kcal)	1632.39 ± 585.06	1615.65 ± 501.16	1617.26 ± 528.41	1604.83 ± 640.68
Total Fat (g)	65.95 ± 29.02	61.82 ± 26.07	66.99 ± 27.59	63.68 ± 31.06
Total SFA (g)	21.28 ± 10.26	20.16 ± 10.02	23.05 ± 12.54	21.70 ± 13.27
Total MUFA (g)	22.67 ± 10.95	20.66 ± 10.51	22.31 ± 10.00	21.01 ± 10.90
Total PUFA (g)	15.39 ± 10.27	14.90 ± 8.25	14.97 ± 7.34	14.82 ± 7.35
PUFA 18:2 (g)	13.40 ± 8.94	13.11 ± 7.40	13.15 ± 6.36	12.97 ± 6.47
PUFA 18:3 (g)	1.59 ± 1.20	1.43 ± 1.01	1.42 ± 0.81	1.50 ± 0.87
PUFA 18:4 (g)	0.01 ± 0.04	0.01 ± 0.04	0.01 ± 0.04	0.01 ± 0.03
PUFA 20:4 (g)	0.16 ± 0.22	0.15 ± 0.26	0.13 ± 0.09	0.10 ± 0.09
PUFA 20:5 n-3 (EPA) (g)	0.04 ± 0.13	0.03 ± 0.14	0.04 ± 0.14	0.04 ± 0.12
PUFA 22:5 n-3 (DPA) (g)	0.03 ± 0.03	0.02 ± 0.03	0.04 ± 0.08	0.03 ± 0.07
PUFA 22:6 n-3 (DHA) (g)	0.08 ± 0.29	0.07 ± 0.29	0.08 ± 0.29	0.09 ± 0.26
Omega-3 Fatty Acids (g)	1.70 ± 1.41	1.53 ± 1.07	1.56 ± 1.07	1.65 ± 1.02
PUFA 18:3 n-3 (ALA) (g)	1.54 ± 1.21	1.40 ± 1.01	1.40 ± 0.81	1.48 ± 0.86
Solid Fats (g)	26.50 ± 15.18	25.99 ± 17.65	30.60 ± 21.34	27.88 ± 21.05
PUFA 18:2 n-6 (LA) (g)	13.05 ± 8.94	12.75 ± 7.37	12.80 ± 6.36	12.62 ± 6.40
PUFA 18:3 n-6 (GLA) (g)	0.01 ± 0.01	0.01 ± 0.01	0.01 ± 0.02	0.01 ± 0.02
PUFA 20:4 n-6 (AA) (g)	0.15 ± 0.22	0.14 ± 0.26	0.11 ± 0.09	0.09 ± 0.09
Omega-6 Fatty Acids (g)	13.20 ± 8.99	12.90 ± 7.35	12.93 ± 6.40	12.72 ± 6.44

Data are presented as the group mean for each dietary intake (average intake per day) assessed by group (Intervention, n = 22; Placebo, n = 22).

Abbreviations: Standard Deviation, SD; Saturated Fatty Acids, SFA; Monounsaturated Fatty Acids, MUFA; Polyunsaturated Fatty Acids, PUFA; eicosapentaenoic acid, EPA; docosapentaenoic acid, DPA; docosahexaenoic acid, DHA; alpha-linolenic acid, ALA; linoleic acid, LA; gamma-linolenic acid, GLA; arachidonic acid, AA
